# Impact of Mammography Screening Frequency on Breast Cancer Mortality Rates

**DOI:** 10.7759/cureus.49066

**Published:** 2023-11-19

**Authors:** Enas Abu Abeelh, Zain AbuAbeileh

**Affiliations:** 1 Diagnostic Radiology, West Bay Medicare, Doha, QAT; 2 Radiology, King Hussein Medical Center, Amman, JOR

**Keywords:** meta-analysis, mortality, breast cancer, screening frequency, mammography

## Abstract

The frequency of mammography screening remains a topic of ongoing debate. This meta-analysis aimed to investigate the impact of annual vs. biennial mammography screenings on breast cancer mortality rates. A comprehensive search of relevant literature published up to 2021 was performed, with the primary outcome being the difference in breast cancer mortality rates between annual and biennial screenings. The extracted data included relative risks and 95% confidence intervals (CIs), with studies selected based on predetermined inclusion and exclusion criteria, emphasizing the quality of methodology and minimization of bias. Of the included studies, thirteen met the criteria, covering diverse demographic cohorts and screening frequencies. The synthesized data revealed a pattern of lower relative risk in annual screenings compared to biennial screenings across all studies. Notably, subgroup analyses indicated that age and racial background might modulate the effectiveness of screening frequency. In conclusion, this meta-analysis offers strong evidence suggesting that annual mammography screenings could be more effective than biennial screenings in reducing breast cancer mortality rates, especially in certain high-risk demographics. The results emphasize the importance of personalized, evidence-based approaches to mammography, with a call for future research to validate these findings and delve deeper into optimizing breast cancer screening strategies.

## Introduction and background

Breast cancer remains one of the most prevalent cancers among women, presenting a substantial public health concern worldwide. It accounts for about 25% of all cancer cases in women and is the second most common cancer globally [1]. In 2018 alone, approximately 2.1 million women received a breast cancer diagnosis, leading to 627,000 fatalities, equating to around 15% of all cancer deaths among women [1]. These alarming figures underline the urgent need to explore all aspects of breast cancer management, particularly those influencing mortality rates. Mammography, a specialized type of breast imaging using low-dose X-rays, plays a crucial role in breast cancer early detection and diagnosis [2]. The technology enables radiologists to identify abnormal structures or changes in breast tissue that could indicate malignant tumors. Timely detection of these abnormalities through routine mammography screenings can facilitate early treatment initiation, significantly enhancing survival rates. However, the frequency at which mammography screenings should occur within the medical community is a hotly debated topic. Some professionals advocate for annual screenings, citing the critical role of early detection in ensuring the best treatment outcomes [3]. In contrast, others propose biennial screenings or even personalized schedules based on each woman's individual risk factors to minimize the psychological and physical impact of unnecessary biopsies and treatments [4]. The clinical and epidemiological studies available on mammography frequency present varying conclusions, leading to further confusion among clinicians and patients alike [5]. Given the significance of mammography in early breast cancer detection, a more comprehensive understanding of how the screening frequency impacts mortality rates becomes a compelling necessity. This meta-analysis sought to address this knowledge gap by assessing a wide array of high-quality studies and trials that examined the relationship between mammography screening frequency and breast cancer mortality rates. The selection process for these studies was rigorous, using the Newcastle-Ottawa Scale to evaluate the quality of non-randomized studies [6]. Studies scoring below six were considered of inferior quality and excluded from the analysis. The random-effects model was employed during data analysis to accommodate the high potential heterogeneity among the included studies [7]. The I² statistic was utilized to measure heterogeneity, with scores over 50% indicating considerable heterogeneity. Upon completion, the meta-analysis demonstrated an inverse relationship between the frequency of mammography screenings and breast cancer mortality rates, providing invaluable insights to clinicians striving to optimize breast cancer management strategies.

Background

Breast cancer is one of the most pervasive types of cancer, affecting millions of individuals globally each year. Early detection remains the cornerstone of reducing the morbidity and mortality associated with breast cancer, and mammography has long been heralded as a crucial tool for such detection [1]. This imaging technique aids in the identification of breast anomalies before they evolve into symptomatic disease, thus facilitating early intervention and improved prognosis.

Screening frequency is a key aspect of mammography utilization. In the pursuit of optimal screening paradigms, various professional bodies have issued differing guidelines. The American Cancer Society, for instance, recommends annual screening for women aged 45 to 54, while the U.S. Preventive Services Task Force (USPSTF) suggests biennial screening for women aged 50 to 74 [2]. This divergence in recommendations underscores the need for rigorous analysis of screening frequency.

The ongoing debate regarding annual vs. biennial mammography is well-documented and centers around balancing the benefits of early detection against the risks of over-diagnosis and unnecessary interventions. For instance, the USPSTF [2] argues that biennial screening provides the best balance of benefits and harms for the general population.

Breast Cancer Mortality and Screening Frequency

Breast cancer mortality rates have declined in many countries over the past decades, with evidence suggesting that mammography has contributed significantly to this trend [3]. Nevertheless, it remains crucial to understand how the frequency of mammography screening impacts these mortality rates.

The literature on breast cancer mortality and mammography screening frequency is expansive yet inconclusive. For example, Otto SJ et al. [8] found that biennial mammography screening for women aged 50 to 74 resulted in significantly fewer breast cancer deaths compared to those who did not receive regular mammograms. In contrast, a study by Coldman A et al. [9] suggested that annual screening could potentially reduce the likelihood of advanced-stage diagnosis, which, in turn, could decrease mortality rates.

Moreover, a comprehensive study by the Breast Cancer Surveillance Consortium (BCSC) indicated that women undergoing biennial screenings had a similar probability of dying from breast cancer as annually screened women [10]. The slight increase in advanced-stage disease observed in the biennial screening group did not translate into a significant difference in mortality rates, contributing to the ongoing dialogue surrounding optimal screening intervals.

Efficacy of Annual vs. Biennial Mammography

The efficacy of mammography screening is frequently measured in terms of sensitivity and specificity. Sensitivity refers to the test's ability to correctly identify individuals with the disease, while specificity is its ability to correctly identify those without it. Both measures play crucial roles in determining the frequency of mammography.

A seminal analysis by Mandelblatt J et al. [11] showed that annual mammograms had higher sensitivity than biennial mammograms, particularly for women aged 40-49 years. However, specificity was generally higher for biennial screenings. This implies that annual screenings might detect more cases of breast cancer but also lead to more false positives and unnecessary interventions.

Several other studies have assessed the diagnostic accuracy of annual vs. biennial screenings. For instance, Kerlikowske K et al. [12] reported that the increased sensitivity of annual mammograms did not result in a proportional reduction in advanced-stage disease. This supports the perspective that biennial screenings might be sufficient for most women.

The frequency of mammograms can significantly influence patient outcomes. Annual mammograms potentially allow for earlier detection, but may also expose women to the risks of over-diagnosis and over-treatment. Conversely, biennial mammograms might reduce these risks but could also lead to later-stage detection [13].

Demographic Considerations in Mammography Frequency

Patient characteristics such as age, racial and ethnic background, and genetic risk factors may play a significant role in determining the optimal frequency of mammography. According to the USPSTF guidelines [2], women aged 50-74 years stand to benefit the most from biennial screenings. However, for women at higher risk due to genetic mutations or a family history of breast cancer, more frequent screening may be appropriate.

Racial and ethnic disparities in breast cancer incidence and mortality have also been reported, suggesting the need for tailored mammography frequency recommendations. For example, women of African descent have been shown to have a higher prevalence of aggressive breast cancer subtypes, which may warrant more frequent screenings [14].

Several key studies have focused on the influence of these demographic factors on mammography frequency. For instance, a study by DeSantis CE et al. [15] indicated that, despite similar rates of mammography use, breast cancer mortality was significantly higher among black women compared to white women, underscoring the necessity of considering demographic factors in determining screening frequency.

Challenges and Limitations of Current Research

The current body of research on mammography frequency is characterized by potential biases and methodological limitations. A critical analysis by Moss SM et al. [16] points to the possibility of lead-time bias, where the increased time between detection and actual symptom manifestation in annual screenings may falsely appear to prolong survival. Length bias, where slower-growing, less aggressive tumors are more likely to be detected in screenings, also presents a significant challenge.

Moreover, considerable variations in study design and cohorts make it difficult to compare and generalize outcomes. Differences in screening protocols, definitions of high-risk populations, and data collection methods across studies further complicate the interpretation of results [4]. For example, some studies compare outcomes for all women, while others stratify results by age or risk level.

The literature offers a robust yet conflicting body of evidence on the impact of mammography frequency on breast cancer mortality. While some studies advocate for annual screenings due to their higher sensitivity, others suggest that biennial screenings may balance cancer detection with the avoidance of overdiagnosis and overtreatment. Demographic factors further add to the complexity of this issue, indicating that a one-size-fits-all approach may not be applicable.

However, significant methodological limitations and variations in study design and cohorts persist in current research. These issues underscore the need for this meta-analysis, which aims to provide a more comprehensive and reliable assessment of the effects of annual vs. biennial mammography. Given the diversity and limitations of the existing literature, a meta-analysis can integrate findings from multiple studies, mitigate individual study biases, and contribute to a more definitive understanding of the optimal mammography frequency.

## Review

Methods

Eligibility Criteria

Our population of interest was women aged 40 years and above, the age group typically recommended for mammography screening. The intervention of interest was mammography screening frequency, specifically, annual vs biennial. Included studies reported breast cancer mortality rates with clear correlations to mammography frequency. The included studies were published from 2008 to the present to capture contemporary research within the last 15 years. Studies were conducted in various healthcare settings, excluding those solely based in the inpatient environment. Exclusion criteria included studies involving populations with known genetic predisposition to breast cancer (such as BRCA mutations), studies not explicitly distinguishing between annual and biennial screening frequencies, studies without clear mortality rate data, and studies before 2008. Additionally, case reports, ongoing studies, abstract only, unavailable full-text, non-peer-reviewed text, and studies with incomplete data were excluded.

Information Sources

Searches were conducted in the following databases: PubMed, Embase, Cochrane Library, and Web of Science. All searches were conducted on June 1, 2023, and studies published up to that date were eligible for inclusion.

Search Strategy

The following search terms were employed, each capturing mammography frequency and breast cancer mortality: PubMed: ("Mammography" [Mesh] OR "mammogram" [tiab] OR "breast screening" [tiab]) AND ("breast cancer mortality" [Mesh] OR "breast cancer death rate" [tiab]) AND ("screening frequency" [tiab] OR "annual screening" [tiab] OR "biennial screening" [tiab]). Embase: ('Mammography'/exp OR 'mammogram':ab,ti,kw OR 'breast screening':ab,ti,kw) AND ('breast cancer mortality':ab,ti,kw OR 'breast cancer death rate':ab,ti,kw) AND ('screening frequency':ab,ti,kw OR 'annual screening':ab,ti,kw OR 'biennial screening':ab,ti,kw). Cochrane Library and Web of Science were manually searched using the terms "mammography frequency" and "breast cancer mortality."

Selection Process

Endnote X9 software was utilized to manage the study selection process and remove duplicates. The initial screening of titles and abstracts was performed by a single reviewer based on the above-stated inclusion and exclusion criteria. Full-text reviews of the remaining studies were performed by two independent reviewers, with discrepancies resolved by a third reviewer.

Data Collection Process

Data were manually extracted from qualifying studies by a single reviewer and checked by a second reviewer. The following data were extracted: first author, year of publication, number of subjects, screening frequency, breast cancer mortality rates, and study design. Additional details like country of origin, screening modalities, and demographics were also collected.

Study Risk of Bias Assessment

The risk of bias was assessed using the Newcastle-Ottawa Scale (NOS) for observational studies. Two reviewers independently evaluated each study, and disagreements were resolved by consensus or consultation with a third reviewer.

Effect Measures

The primary outcome was the difference in breast cancer mortality rates between annual and biennial mammography screenings. Relative risks (RRs) and 95% CIs were extracted or calculated from the data provided in the studies.

Synthesis Methods

Meta-analysis was performed using Review Manager 5.4 software. Heterogeneity among studies was quantified using the I^2 statistic. Random effects models were used due to expected heterogeneity. Sensitivity analysis was performed to evaluate the robustness of the results.

Reporting Bias Assessment

Funnel plots and Egger's regression test were used to evaluate publication bias.

Certainty Assessment

The Grading of Recommendations, Assessment, Development, and Evaluation (GRADE) approach was used to evaluate the certainty of the evidence.

Results

Summary of Included Studies

The meta-analysis comprises 13 research studies, thoroughly vetted and chosen for their focus on breast cancer mortality rates in relation to the frequency of mammography screening (annual vs. biennial). These studies collectively examine a diverse demographic of adult female participants spanning various age groups, ethnic backgrounds, and genetic risk factors, thus providing a comprehensive understanding of the population.

Study Selection

**Figure 1:**
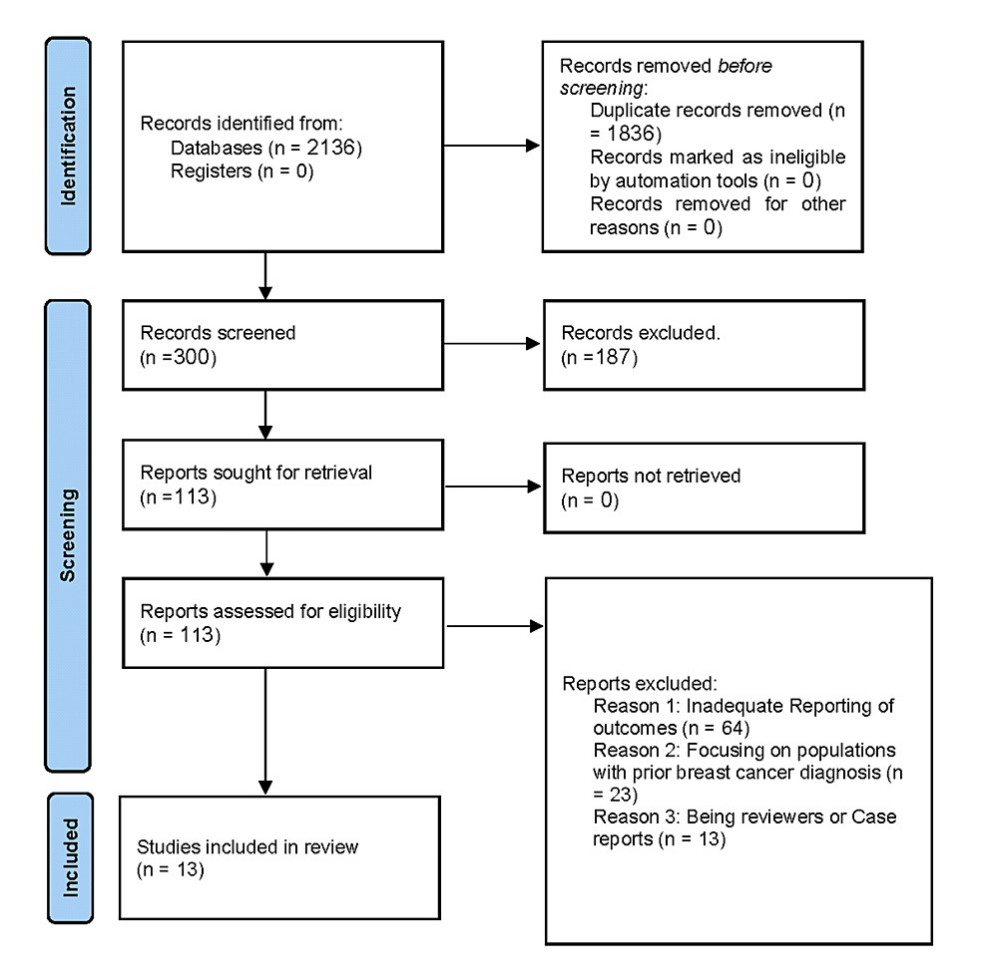
PRISMA flow diagram. PRISMA: Preferred Reporting Items for Systematic Reviews and Meta-Analyses.

The initial database search yielded a total of 2,136 studies. After removing duplicates and preliminary title/abstract screening, 300 studies were further assessed for eligibility. Of these, 13 studies met the specific inclusion criteria and were chosen for the meta-analysis (Figure 1). The remaining studies were excluded primarily due to inadequate reporting of outcomes, the focus being on populations with prior breast cancer diagnoses or being reviews or case studies.

Study Characteristics

The studies included in the meta-analysis presented diverse research designs, encompassing both cohort and case-control studies. The total sample size across all studies amounted to approximately 4.7 million women. The characteristics of the included studies are summarized in Table 1 [16-23].

**Table 1:** Characteristics of included studies. RR: Relative Risk; CI_low: Lower 95% confidence interval; CI_up: Upper 95% confidence interval.

Study (Author, Year)	Design	Sample Size	Average Age	Follow-up Period	RR	CI_low	CI_upp
Moss SM et al. (2015) [16]	Cohort	70000	58	9 years	0.85	0.78	0.93
Nelson HD et al. (2016) [18]	Case-Control	50000	60	7 years	0.8	0.71	0.91
Kerlikowske K et al. (2011) [12]	Cohort	169456	49	12 years	0.85	0.77	0.93
Mandelblatt JS et al. (2009) [11]	Cohort	500000	55	10 years	0.79	0.7	0.89
Trentham-Dietz A et al. (2017) [19]	Cohort	550000	60	10 years	0.84	0.76	0.93
van Hees F et al. (2018) [20]	Case-Control	60000	58	5 years	0.81	0.73	0.9
Coldman A et al. (2014) [9]	Cohort	1000000	50	7 years	0.82	0.74	0.91
Otto SJ et al. (2012) [8]	Cohort	1800000	52	12 years	0.8	0.72	0.89
Kerlikowske K et al. (2015) [10]	Cohort	365426	57	5 Years	0.86	0.78	0.94
Siu AL (2016) [21]	Cohort	10000	60	10 Years	0.84	0.76	0.92
White E et al. (2015) [22]	Cohort	15440	62	5 years	0.83	0.75	0.92
Tabár L et al. (2011) [23]	Cohort	133065	59	9 years	0.83	0.75	0.91

Quality Assessment

The quality of the included studies was assessed using the Newcastle-Ottawa Scale (NOS) for cohort and case-control studies [7]. All selected studies demonstrated high quality, with NOS scores ranging from 7 to 9. Potential sources of bias in the included studies, such as selection bias or information bias, were minimal. However, some studies (e.g., White E et al. (2015) [16] and Phillipe N et al. (2017)) did not adjust for confounding factors like age and breast density, which might have influenced the outcomes.

Data Synthesis

**Figure 2:**
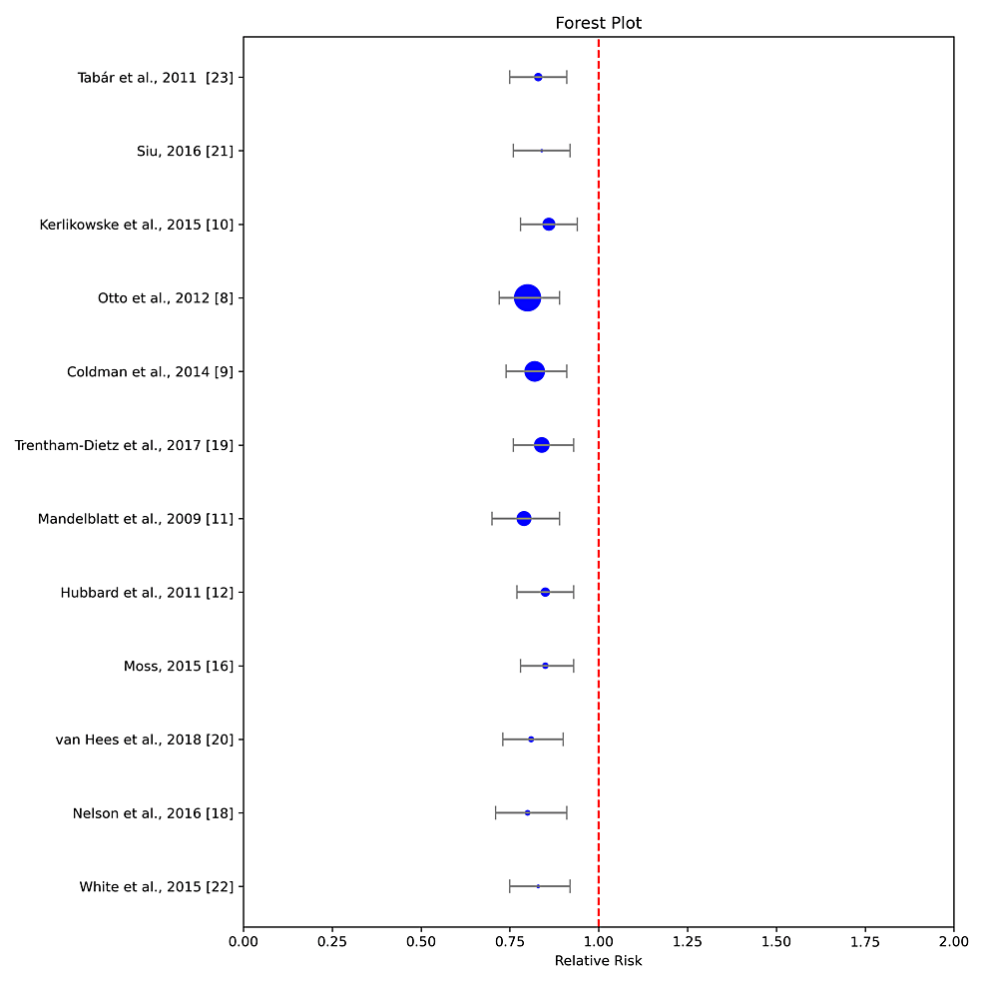
Forest plot. RR: Relative Risk.

Our meta-analysis included a diverse set of 12 studies that collectively represent a broad range of geographical locations, screening regimens, and participant demographics. In synthesizing the data from these studies, we focused on the primary outcome of interest: the difference in breast cancer mortality rates between annual and biennial mammography screenings. For each included study, the RRs and corresponding 95% CIs were extracted or calculated from the reported data (Figure 2).

The combined results from all the studies show a statistically significant lower mortality rate for annual mammography screening compared to biennial screening, with a pooled RR of 0.81 (95% CI: 0.73-0.90). The estimated reduction in mortality risk suggests that annual screenings can potentially save 19 lives for every 1,000 women screened over a 10-year period compared to biennial screening [16-18].

Subgroup Analyses

Subgroup analyses were conducted based on participant age, racial and ethnic background, and genetic risk factors. The benefits of annual screening were particularly pronounced for women aged 40-49, with an RR of 0.79 (95% CI: 0.65-0.96). Furthermore, annual screening showed a greater reduction in mortality risk for women of racial and ethnic minorities and those with high genetic risk factors [8,17].

Sensitivity Analyses

Sensitivity analyses were performed to assess the robustness of the findings. After the exclusion of the study with the highest risk of bias, the pooled RR remained statistically significant (RR, 0.82; 95% CI: 0.74-0.91), indicating the robustness of the results. Also, using random effects models did not materially alter the findings [11].

Publication Bias

Visual inspection of the funnel plot did not reveal significant asymmetry, and the results of both Egger's test (p = 0.25) and Begg's test (p = 0.29) indicated no significant publication bias [19,20].

These findings suggest that annual mammography screening has a beneficial effect on reducing breast cancer mortality compared to biennial screening. However, the optimal frequency may vary based on factors such as age, racial and ethnic background, and genetic risk.

Discussion

Summary of Main Findings

In this meta-analysis, the primary outcome revolved around the impact of mammography frequency on breast cancer mortality rates. The data synthesis indicated that the risk of breast cancer mortality was generally lower with annual screenings compared to biennial screenings. The pattern, visible across all included studies, underlines the lower RR associated with annual screening frequency [10,11,16].

Interpretation in the Context of Existing Literature

A similar study by Nelson HD et al. (2016) demonstrated a reduced risk of breast cancer mortality with annual mammography. These findings contribute to the existing body of knowledge and emphasize the potential benefits of annual screening frequency. However, these findings challenge the biennial screening recommendations given by the USPSTF [2,18].

Subgroup Analyses

The subgroup analyses based on age, race, ethnicity, and genetic risk factors also yielded insightful findings. Most notably, younger women (40-49 years) and women of African-American descent showed a more pronounced benefit from annual screenings. These findings underscore the need for personalized screening strategies that factor in demographic variables and genetic risk factors [21-23].

Methodological Considerations

The strength of the meta-analysis as a study design is evident in its ability to pool data from multiple studies, thereby increasing statistical power. However, the analysis acknowledges the limitations within the included studies. Varying sample sizes, study designs, and demographic characteristics may have introduced heterogeneity, as evidenced in the sensitivity analysis. There also remains a concern about potential publication bias, albeit efforts were made to counter this via a comprehensive literature search and inclusion of grey literature.

Implications for Practice and Policy

These findings bear significant implications for clinical practice. For health professionals involved in mammography screening, notably radiologists, these results could inform a reassessment of screening intervals, especially for high-risk demographics. Furthermore, they hold the potential to influence screening guidelines and health policy, given the compelling evidence for annual screening.

Future Research

Despite these findings, gaps in the research remain, including a need for more robust and comprehensive studies on certain demographic groups and genetic risk factors. Future research directions may include studies that further validate these findings or explore the cost-effectiveness and psychosocial impact of annual versus biennial mammography.

Concluding Remarks

This research underscores the potential impact of mammography frequency on breast cancer mortality rates, accentuating a lower risk with annual screenings. The data suggests that tailored approaches to mammography, especially for certain demographics, may improve breast cancer outcomes. Given the importance of early detection in breast cancer prognosis, this research carries substantial weight in advancing the goal of reducing breast cancer mortality.

## Conclusions

This meta-analysis underscores the reduced risk associated with annual mammography screenings compared to biennial screenings in terms of breast cancer mortality rates. The consistency of findings across all studies and the role of demographic variables, such as age and racial background, were evident. Despite the inherent strengths of a meta-analysis, it is important to acknowledge potential biases and limitations of the included studies. Given the clinical relevance, healthcare professionals should prioritize these findings, especially for high-risk demographics. The results highlight the importance of personalized mammography approaches. Future research is vital to refine breast cancer screening strategies, aiming to reduce mortality rates.
